# Nasal valve obstruction: a comprehensive analysis of the current literature and proposal of a management algorithm

**DOI:** 10.3389/fsurg.2025.1549915

**Published:** 2025-03-27

**Authors:** Francesca Pirola, Raymond Kim, Richard Douglas

**Affiliations:** ^1^ ORL Department, Auckland City Hospital, Auckland, New Zealand; ^2^ Department of Surgery, Faculty of Medical and Health Sciences, University of Auckland, Auckland, New Zealand

**Keywords:** nasal valve, valve collapse, nasal obstruction, nasal obstruction measurement, nasal valve repair

## Abstract

Nasal valve obstruction (NVO) remains challenging to diagnose and treat. This review explores the persisting controversies of NVO management, emphasising the lack of consensus in diagnostic criteria and treatment protocols among otolaryngologists and facial plastic surgeons. Recent surveys highlight the central dilemma: for many patients who have both septal deviation and NVO, a septoplasty alone provides adequate symptomatic improvement. However, some with this combination of problems continue to be troubled by nasal obstruction if only a septoplasty is performed. This underscores the critical question of when NVO necessitates specific surgical intervention. The varying diagnostic approaches that have been used and the limited outcome data currently available conspire to make this question difficult to answer with certainty. This review will also address the role of the inferior turbinates in NVO management, proposing their inclusion in NVO surgical planning. Other techniques will also be discussed for their potential impact on nasal valve dynamics. Both endonasal and open approaches to correcting the caudal septum need to be considered when discussing NVO repair. A treatment algorithm for NVO will be presented as a practical guide for the management of this condition.

## 1 Introduction

**Figure 1:**
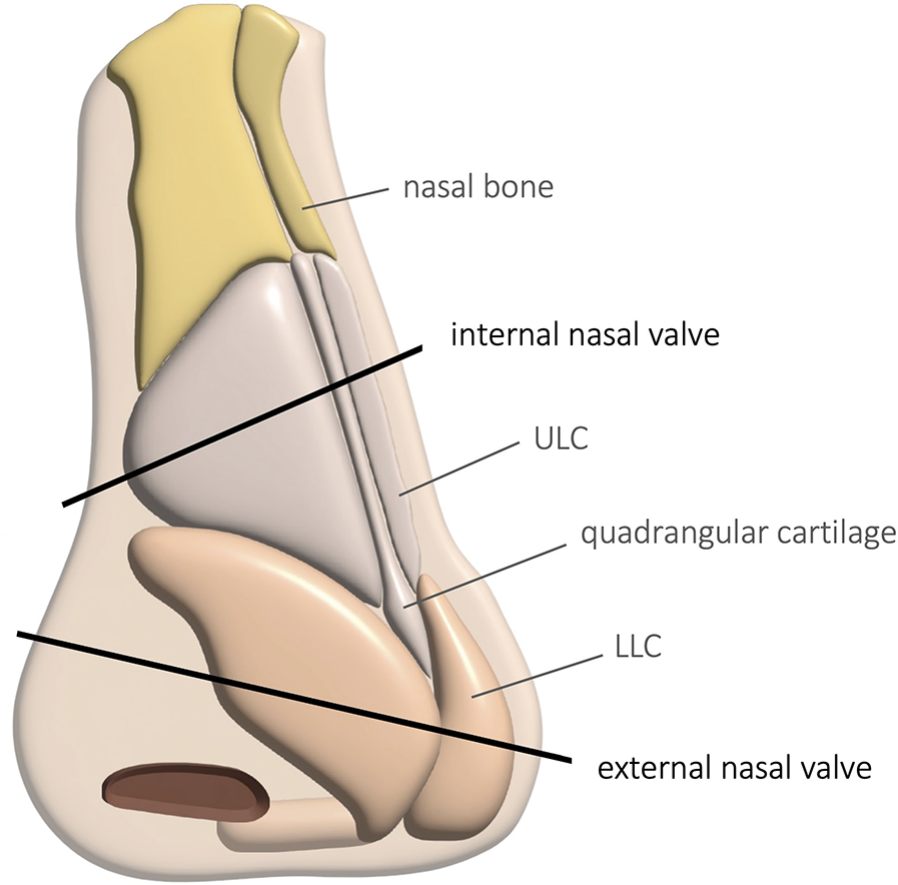
Outer anatomy of the nose and localization of the internal and external nasal valves. (ULC, upper lateral cartilage; LLC, lower lateral cartilage).

Nasal valve obstruction (NVO) is defined as a reduction in airflow secondary to dysfunction of the nasal valve, where minor anatomical variations can substantially elevate nasal resistance. Boundaries of the nasal valve are the nasal septum medially, the upper (ULC) and lower lateral cartilages (LLC) superolaterally, the head of the inferior turbinate (HIT) inferolaterally, and the nasal floor inferiorly. The nasal valve may be further divided into external and internal valves (Figure 1). The external nasal valve (ENV) is recognized as that part of the lateral wall that collapses on forced inspiration. Its lateral wall is composed largely of the lower lateral cartilage and surrounding soft tissues of the nasal ala. The caudal septum is the medial wall, and the vestibule forms the floor. The internal nasal valve (INV) is the narrowest part of the nasal passage, and as such, it is responsible for the greatest proportion of nasal resistance to airflow. Unlike external valve collapse, narrowing of the internal valve is usually not apparent on forceful inspiration, but rather, its diagnosis requires a direct examination of the nasal passage. The key anatomical component of the internal valve is the angle formed between the upper lateral cartilage and the septum. This overlapping region between the external and the internal nasal valve is the “scroll” area, where the upper and lower cartilages connect with each other through connective tissue. This area is fundamental to nasal valve stability. Enlargement of the head of the inferior turbinate, usually in response to an inflammatory condition or due to bony hypertrophy, can also significantly influence the cross-sectional narrowing of this region.

**Table 1:** Representative literature on nasal valve collapse: study characteristics and outcomes.

Authors	Year	Study design	Sample size (n)	Materials & methods	Results	Main findings
Diagnostic tools for NVO						
Barnes ML, Lipworth BJ	2007	cross-sectional	74	Examined PNIF changes with two different nasal stents	Improvement in PNIF was 1.7 L/min (*p* *=* 0.42) with Nozovents and 25.4 L/min (*p* < .001) with Sinuscones	PNIF is a useful objective measure for nasal valve obstruction
Bonaparte JP, Campbell R	2018	prospective cohort	141	Evaluated the utility of the Cottle maneuver in predicting septoplasty outcomes. Follow-up at 12 months with NOSE score	71.5% of patients: positive Cottle maneuver at baseline. No significant correlation between pre-op Cottle maneuver and post-op improvement (*p* = 0.38).	Cottle maneuver has limited predictive value in nasal septal surgery
Das A, Spiegel JH	2020	cross-sectional	100	Assessed validity of the Cottle maneuver in diagnosis of NVO	98% of subjects reported improvement with Cottle maneuver, regardless of anatomical obstruction	Cottle maneuver lacks specificity for NVO diagnosis
Fung E et al.	2014	prospective cohort	35	Assessed validity of the modified Cottle maneuver to identify INV vs. ENV collapse. Outcome measures: VAS and ROE scores	24 with ENV collapse received batten grafts 11 with INV collapse received spreader grafts ROE score increased (*p* < 0.001) VAS for satisfying nasal breathing increased (*p* < 0.001) Spearman rank correlation was strong for INV (rho = 0.80; *p* < 0.05) and moderate for ENV (rho = 0.50; *p* < 0.05)	Modified Cottle maneuver can be effective in predicting positive surgical outcomes, distinguishing between INV and ENV collapse to help select the appropriate surgical treatment
Gagnieur P et al.	2022	prospective cohort	66	Assessed four-phase rhinomanometry for INV collapse diagnosis	Sensitivity 88.3% and specificity 89.9% for diagnosis of INV collapse, reproducing clinical diagnosis	Rhinomanometry is a useful tool for diagnosing INV collapse
Rimmer J et al.	2019	narrative review	32	Reviewed objective diagnostic tools for NVO	Acoustic rhinometry and rhinomanometry most reliable; Cottle maneuver least specific	Objective airflow assessment is essential for NVO diagnosis
Expert consensus and guidelines on NVO						
Rhee JS et al.	2010	clinical consensus	8	Review of NVO diagnosis and treatment by a panel of 8 surgeons (FPRS and ORL)	NVO is best evaluated with history and physical examination. Radiographic studies are not useful. Other objective nasal outcome measures may not be useful or accepted for NVO. Nasal steroids are not useful for NVO unless rhinitis is present. Nasal strips, stents, or cones can be beneficial for NVO in some patients. Surgery is the primary mode of treatment for NVO. Septoplasty and/or turbinate surgery can treat NVO. Surgery to support the lateral nasal wall/alar rim is a distinct entity and can be necessary	Established consensus on diagnostic and treatment guidelines
Wang Y, Bonaparte JP	2019	survey study	86	NVO diagnosis and treatment patterns among Canadian ORL surgeons was assessed via a 20-item survey	Inconsistent tools used to diagnose NVO among participants. The reportedly most important diagnostic tools were: Cottle maneuver (62.8% for ENV), visual inspection (39.5% for INV), modified Cottle maneuver (25.6% in INV), failed septoplasty (11% for ENV, 7% in INV)	Significant heterogeneity in diagnosing NVO and in the opinions on treatment indications and methods
Treatment strategies for NVO						
André RF, Vuyk HD	2008	prospective cohort	84	Investigated butterfly graft technique for INV incompetence both in primary and revision cases	Subjective nasal airflow improved in 93% of cases	Butterfly graft effectively widens INV angle and stabilizes nasal valve
André RF, Vuyk HD	2008	prospective cohort	33	Investigated nasal valve suspension technique outcomes. Patients rated subjective improvement on 1–10 VAS	Unchanged: 7 (21%) Improvement (VAS 1–3): 17 (52%) Improvement (VAS ≥4): 9 (27%) Average improvement: 2.3/10 on VAS scale Complications: 9/33	Nasal valve suspension technique is not recommended by the authors
Erickson B et al.	2016	prospective cohort	17	AR and nasal endoscopy to assess INV function after septoplasty + IT reduction + spreader grafts. Outcome measures: NOSE, SNOT-22, validated endoscopic grading of INV collapse	INV collapse grading, MCA, NOSE and SNOT-22 scores improved	Spreader grafts significantly improve nasal airflow and INV stability. Endoscopic grading of INV collapse has moderate inter-rater and intra-rater reliability.
Goudakos JK et al.	2016	systematic review	53 studies	Literature search across multiple databases (MEDLINE, EMBASE, Cochrane Library)	No significant difference in outcomes for various techniques. Low-level evidence with 50 studies rated as level IV evidence. Studies primarily described surgical techniques	No significant conclusions on the superiority of one technique over another
Schlosser RJ, Park SS	1999	cadaver study and retrospective cohort	6 cadavers 35 patients	Measurement of MCA after spreader grafts, flaring sutures, and both Assessment of nasal patency and outcomes post-surgery with VAS score	Increase in MCA: Spreader grafts: 5.4% Flaring sutures: 9.1% Combined: 18.7% (*p* < .05) Spreader grafts in 21/35 (60%),+flaring sutures in 20/21 Flaring sutures: 28/35 Alar batten grafts: 17/35 Significant improvement in nasal function (*p* < .01)	Combining spreader grafts and flaring sutures provides the best improvement in nasal valve function. Either technique, used alone, failed to have a statistically significant impact on the minimal cross-sectional area of the nasal valve
Green A et al.	2023	retrospective cohort	13,895	Evaluated the effectiveness of septoplasty in NVO patients	1.9% required revision surgery with further techniques to support the lateral walls of the nasal valve 23.1% of revision procedures required extranasal cartilage graft	Prior septoplasty increases risk of requiring extranasal cartilage graft if subsequent valve repair is necessary. Patients with NVO would benefit from upfront complete treatment addressing septum and nasal valve
Rhee SJ et al.	2008	systematic review	44	Systematic review of the literature (1982–2007 PubMed) of articles discussing functional rhinoplasty and valve repair for NVO. Study design, interventions, outcomes, and level of evidence were assessed. Data extraction included subjective and objective outcome measures, and objective tests	42/44 studies classified as level 4 evidence (case series) 2/44 studies classified as level 2b (cohort studies) Various surgical techniques were used: spreader grafts, alar batten grafts, butterfly onlay grafts, and structural bone grafting. All studies reported improvement post-surgery (effectiveness rates from 65% to 100%). Most common complications: failure to improve airway patency, intranasal synechiae (14%), infection (9%), graft resorption (7%), septal perforation (7%). Septoplasty and turbinate reduction were commonly performed alongside nasal valve repair.	There is substantial level 4 evidence supporting the effectiveness of functional rhinoplasty and nasal valve repair in treating NVO. The lack of high-level evidence (randomized controlled trials) limits the strength of conclusions. No comprehensive and uniform strategy to approach nasal valve dysfunction can be deduced.
Silvers SL et al.	2021	RCT	118	Evaluated RF treatment (VivAer) for NVO. Responder rate at 3 months using NOSE score	77 patients (treatment arm) received RF with VivAer 41 patients (placebo arm) received a sham procedure Success rate 88.3% in treatment arm vs. 42.5% in placebo arm (*p* < .001)	Radiofrequency is safe and effective in treating NVO in short-term follow-up
Simmons JK et al.	2024	retrospective cohort	37	Evaluated radiofrequency treatment (VivAer) for NVO in patients with prior rhinoplasty. Outcome measure: NOSE score	21/37 (56.8%) successfully treated 50.0% response rate in the 16 who were re-treated	RF with VivAer can effectively alleviate NVO in patients with a history of rhinoplasty or nasal valve repair, offering an alternative to revision surgery
Wilkins SG et al.	2023	retrospective cohort	26	Analyzed complications of bioabsorbable nasal implants from MAUDE database	26 device reports entered between 2017 and 2022 13/26 abscess, implant protrusion 5/26, 3/26 facial pain/discomfort at 1 year, 3/26 failure to absorb. Management of adverse events included antibiotics (*n* = 9), steroid injections (*n* = 4), and explantation (*n* = 20)	Bioabsorbable implants carry risks of complications, however very limited data were available for analysis

AR, acoustic rhinometry; FPRS, facial plastics and reconstructive Surgeons; IT, inferior turbinates; MCA, minimal cross-sectional area; NVO, nasal valve obstruction; PNIF, peak nasal inspiratory flow; QoL, quality of life; RCT, randomized controlled trial; ROE, rhinoplasty outcomes evaluation; RF, radiofrequency; VAS, visual analogue scale.

Despite its clinical significance, NVO remains challenging to evaluate and treat due to the lack of standardized diagnostic criteria and universally accepted treatment protocols. Current diagnostic approaches vary widely, ranging from subjective manoeuvres such as the Cottle test to objective airflow measurements like rhinomanometry and acoustic rhinometry. However, no single method has been proven to be highly sensitive and specific in distinguishing true NVO from other causes of nasal obstruction. Similarly, the optimal treatment strategy remains controversial.

Many patients with NVO undergo septoplasty or inferior turbinate reduction without significant symptomatic relief, highlighting a critical gap in preoperative assessment and patient selection for nasal valve repair. The relationship between abnormal structural support and airflow dynamics further complicates treatment planning, such as in patients with dynamic valve collapse vs. those with fixed anatomical narrowing.

There is a need for a more structured, evidence-based approach to NVO diagnosis and treatment. This review aims to explore the fundamental pathophysiology and biomechanics of NVO and critically assess the current diagnostic methods. Given the lack of a clear decision-making process for clinicians, a management algorithm will be presented to attempt to integrate clinical experience with the available literature.

## 2 Materials and methods

The PubMed database was interrogated using the following free-text terms: “nasal valve collapse”, “nasal valve obstruction”, “alar collapse”, “nasal valve insufficiency”, “management of nasal valve collapse”, “surgery for nasal valve collapse”, “nasal valve repair”. Through this search, 804 papers were identified and then screened, based on the pertinence of their title and date of publication between 1990 and 2024. English-language studies that discussed diagnostic methods for nasal valve collapse and treatment strategies were initially included. The abstracts of 68 papers were reviewed and the available full texts of 31 pertinent articles were further analyzed and included in the review. No systematic search limitations were used.

## 3 Physics and etiology of nasal valve obstruction

The INV has the shape of a triangle, and the angle of the apex is between 10° to 15°. The average cross-sectional area of the INV is only about 40 to 60 mm^2^, and for this reason, it accounts for between half to two-thirds of the total nasal airway resistance (1, 2).

NVO can have many causes, including congenital structural weaknesses and nasal tip ptosis, trauma, rhinoplasty surgery, loss of rigidity of the cartilages, changes in skin tonicity and elasticity due to ageing, increase in weight of the cheek soft tissues, and rarely chronic inflammatory processes such as polyangiitis. Besides the congenital etiology, one of the most common causes seen in clinical practice is excessive approximation of the upper lateral cartilages to the septum after a dorsal hump removal is performed and the resulting open-book deformity is closed.

Bernoulli's principle and Poiseuille's law define the factors that cause valve collapse. Bernoulli's principle ($p_{1}+\frac{1}{2}\rho v_{1}^{2}=p_{2}+\frac{1}{2}\rho v_{2}^{2}$, where *p*, pressure at the level of the valve; ρ, density of air; *v*, velocity of air) states that air flowing through a narrow segment increases its speed, which leads to a decrease in intraluminal pressure. In this context, during deep inhalation, a narrow or inadequately supported lateral wall can collapse inward, blocking the airflow through the nostril. Poiseuille's law ($R=\frac{8\eta QL}{\pi \;r^{4}}$ where *R* is resistance to airflow and *r* is the radius of the nasal valve area) underlies the reason why even small reductions of the valve width correlate with increasingly higher resistance to airflow.

**Figure 2:**
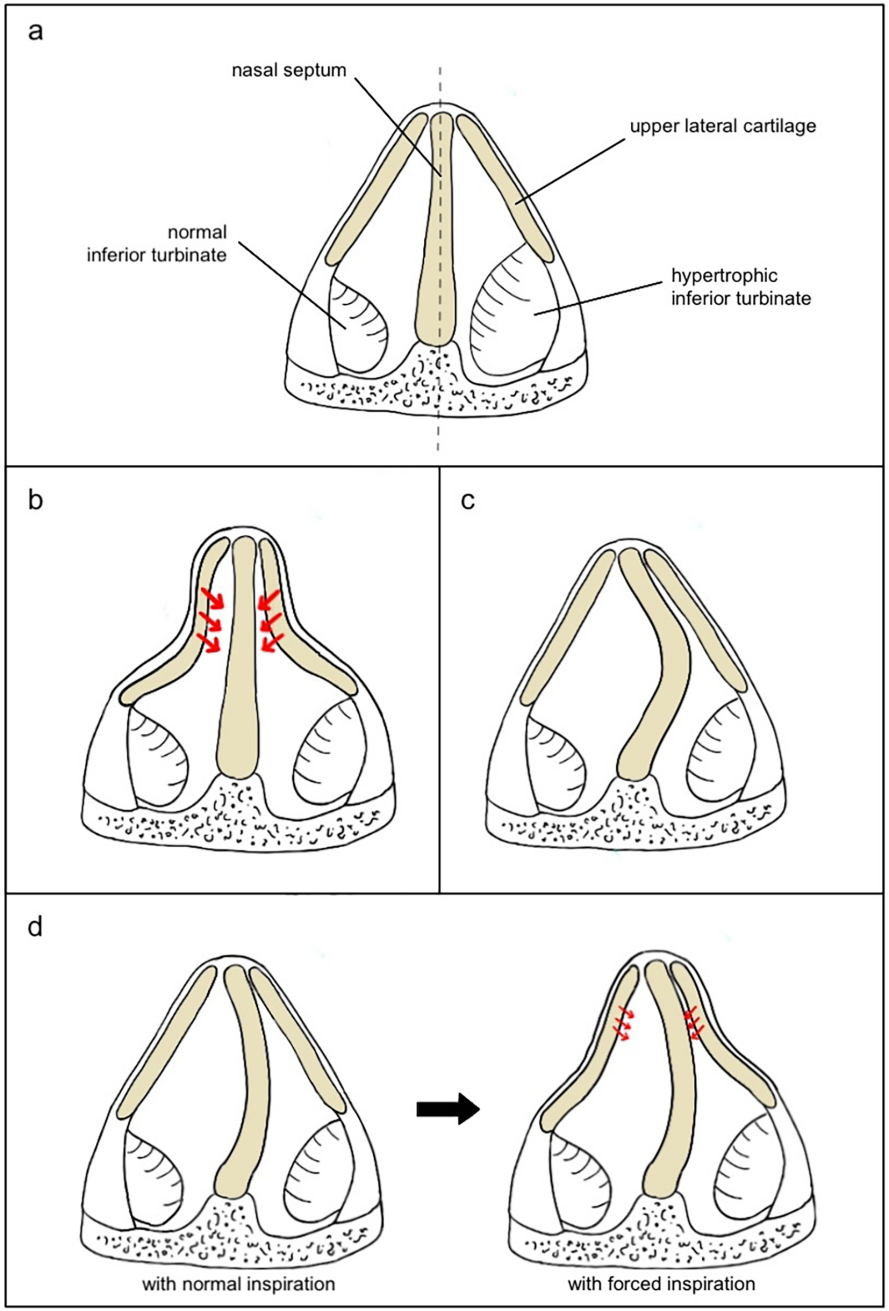
Causes of obstruction at the level of the internal nasal valve (INV). **(a)** INV with normal inferior turbinate (left) vs. hypertrophic inferior turbinate (right); **(b)** INV with straight septum and high collapsibility of the lateral cartilages that cause complete obstruction during inspiration; **(c)** INV with septal deviation and no lateral cartilages collapse; **(d)** INV with mild collapsibility of the lateral walls that do not collapse during normal inspiration (left) but slightly collapse during forced inspiration (right), representing a case that may benefit from septoplasty alone to reduce Bernoulli's effect.

Static and dynamic NVO refer to different mechanisms of obstruction in the nasal valve area, each with distinct characteristics and implications for diagnosis and treatment (Figure 2). Static NVO (SNVO) occurs when the nasal valve is structurally narrow at rest and remains narrow regardless of breathing effort. This is typically due to fixed anatomical issues, such as anterior septal deviations or abnormally shaped upper or lower lateral cartilages. Inferior turbinate enlargement is an important factor to consider. Dynamic NVO (DNVO) occurs when the valve angle is adequate with gentle inspiration, but it becomes increasingly narrow when inspiration is forced. In patients with DNVO, soft tissues of the lateral nasal wall cannot withstand the negative pressure that develops during forced inspiration due to the effect of Bernoulli's principle. Patients may report nasal obstruction that worsens during physical exertion or when taking deep breaths but improves when they breathe normally or at rest.

## 4 Diagnosis of nasal valve collapse

Although the physics behind NVO is understood, the current literature does not present a clear consensus on its diagnosis and management. Precise preoperative assessment of the anatomical causes of NVO facilitates selecting the appropriate therapeutic approach. However, correlating the subjective perception of nasal obstruction with objective measurements of the nasal valve has proven challenging. Various methods have been used to help establish the diagnosis, but none has proven to be entirely satisfactory. An accessible, reliable, and reproducible method to evaluate nasal airflow changes is required for patients with suspected NVO.

The most important first step in examining a patient with nasal obstruction is to observe the shape of the nose, both in quiet and forced inspiration. NVO is much more common in high, narrow (leptorrhine) noses. Deformities may result from previous injury or surgery. Thinning, often due to ageing, of the lateral nasal wall may be apparent, and the collapse of a thin lower lateral cartilage on quiet or moderate inspiration points to a diagnosis of external nasal valve obstruction.

Determining the shape of the anterior part of the septum is a key component of the NVO examination. The caudal edge of the septum may project into the anterior nares, which is often associated with a convexity of the septum on the contralateral side, which can be a potential factor in causing NVO.

Nasal endoscopy is optimally performed with a 3 mm 0° or 30° nasal endoscope, where the latter has the advantage of looking up at the apex angle of the nasal valve. The nostril size and septal shape are noted as the endoscope passes into the nose. Enlargement of the inferior turbinate is a common contributor to NVO because of the high prevalence of rhinitis, and it is recommended to initially examine the nasal valve without applying any decongestants. The size, shape and collapsibility of the lower lateral cartilage may indicate the cause of external valve collapse. Likewise, the upper lateral cartilage and the angle between it and the adjacent septum may indicate that the patient has a narrowing of the internal valve that causes obstruction. The same areas are examined during forced inspiration to assess their collapsibility.

Once the nose has been carefully examined, several maneuvers and investigations can be performed to diagnose NVO.

### 4.1 Cottle's and modified cottle's maneuvers

Cottle's manoeuvre is considered by many to be the answer to diagnosing NVO. Cottle's manoeuvre is performed by distracting the cheek laterally, usually with a couple of fingers, and the modified Cottle's manoeuvre is performed by using an instrument (usually an ear probe) to gently lift the ULC and LLC individually to identify internal or external NVO specifically. However, these tests have been found to have relatively low specificity, as many patients without NVO report an improvement in subjective airway patency while having these manoeuvres performed (3–5).

The effectiveness of the modified Cottle's manoeuvre (MCM) in predicting the outcomes of nasal valve repair was studied in a subset of 45 patients who underwent functional rhinoplasty (batten grafts if external NVO vs. spreader grafts if internal NVO) (6). The patients were asked to describe their unilateral nasal patency on a visual analogue scale before and during the MCM. The manoeuvre was also used to identify the specific site of the nasal valve obstruction. The study showed a significant correlation between the VAS scores with MCM and the postoperative results, supporting the validity of the MCM despite it being a subjective and operator-dependent test. However, another study demonstrated no change in surgical success after septoplasty and turbinate reduction in 170 patients with either positive or negative preoperative Cottle manoeuvres (7). Interestingly, many patients with a negative Cottle manoeuvre presented with perceptible NVO on physical exam, whereas an equal number with a positive Cottle test had no perceptible evidence of NVO. After a one-year follow-up, the authors found that the two most common causes of surgical failure were persistent caudal septal deviation (33%) and static nasal valve narrowing (27%).

According to a consensus statement of the American Academy of Otolaryngology Head and Neck Surgery, the diagnosis of NVO requires both a visible inspiratory collapse of the lateral nasal wall or alar rim and a subjective improvement in nasal airﬂow during CM or MCM (1). Increased nasal obstruction during deep inspiration was also suggestive of NVO. A weak agreement was reached in support of the utility of adult nasal strips (e.g., Breathe Right Strips) for conﬁrming NVO.

Despite the widespread clinical use of the Cottle manoeuvre, the correlation between a positive CM or MCM and the need for nasal valve repair remains weak. A recent study found that 98 of 100 volunteers reported improvement in their nasal breathing with CM or MCM, regardless of their endonasal or nasal valve anatomy (8). This emphasises the low specificity of CM or CMC, which is explained by Poiseuille's law, for which even a slight widening of the nasal valve area dramatically reduces the resistance to airflow. For this reason, several other tests have been utilised to improve diagnostic accuracy.

### 4.2 Peak nasal inspiratory flow

Peak nasal inspiratory flow (PNIF) is a measurement of nasal airflow and patency that uses an airtight facemask over the nose and mouth and records the peak inflow on a monitor. PNIF is measured at maximal nasal inspiration after a full expiration. However, as maximal inspiration may lead to exaggerated nasal valve collapse, PNIF remains a controversial tool. A study investigating the effect of nasal valve dilators showed that PNIF better evaluates how other sites beyond the nasal valve contribute to nasal obstruction (9).

### 4.3 Anterior active rhinomanometry

Anterior active rhinomanometry (AAR) is another objective diagnostic technique that can evaluate nasal airway resistance by measuring the relationship between airflow and pressure during the breathing cycle. AAR is “active” because the patient participates by breathing and “anterior” because measurement tools are placed externally in front of a nasal cavity. The test can independently assess each nostril, allowing for an analysis of unilateral nasal obstruction. Measurements are taken for both nostrils individually, first under normal conditions and then after nasal decongestion, which can help to determine the mucosal contribution to nasal obstruction. The ratio of pressure to airflow is displayed on a pressure-flow curve: the greater the pressure required to generate a certain flow, the more obstructed the airway.

While AAR offers valuable insights into nasal airflow, its accuracy can be influenced by factors such as the patient's inspiratory effort during the test or variations in the nasal cycle, and it does not delineate between the sites of anatomical obstruction without supplementary diagnostic tools (10–12). The devices required are relatively uncommon outside of research laboratories.

### 4.4 Acoustic rhinometry

Acoustic rhinometry is an objective tool that can measure the volume and cross-sectional area of the nasal passage during breath holding. It is a quick and non-invasive technique and does not require patient cooperation beyond remaining still during the measurement. The minimal cross-sectional area (MCA) is the key variable measured: an MCA less than 0.4 cm^2^ has been found to correlate with patients' sensation of nasal obstruction. It is particularly useful for identifying structural causes of nasal obstruction, such as septal deviation, turbinate hypertrophy or static nasal valve collapse (2, 3, 8, 12). However, it has proven hard to define normal values at each level of the nasal cavity, and studies are limited by multiple variables such as mid-facial development, age, weight, factors related to ethnic characteristics, as well as by the specifics of the device used. The room temperature and humidity, as well as the positioning of the sonic sensors, can also contribute to possible causes of errors.

### 4.5 CT scan

Non-contrast enhanced CT scans of the sinuses can be very helpful in the diagnosis of NVO. Not only do they reveal other causes of nasal congestion, such as sinusitis and allergic rhinitis, but also demonstrate the configuration of the septum in three planes and give an objective perception of the size of the inferior turbinates. The shape and size of the upper and lower cartilages, as well as the angle and space between the upper lateral cartilage of the septum, can also be defined.

## 5 Management of nasal valve obstruction

The management of the nasal valve is inherently complex due to its multifactorial causes and the lack of standardised evaluation techniques for nasal valve obstruction. Furthermore, considerations of nasal aesthetics add to the complexity of defining the optimal surgical approach to the nasal valve and tip.

### 5.1 Non-surgical options

Breathing strips and internal nasal dilators are among the most commonly used. Breathing strips are springy, and when adhered to the skin of the nose, they lift the lateral nasal walls to widen the internal nasal valve angle. Internal nasal dilators are inserted into the nostril (unilaterally or bilaterally) to prevent collapse. While these devices are simple to use and generally well tolerated, they are restricted to use during sleep due to their conspicuous nature. If inflammation of the inferior turbinates is significantly causative, treatment with topical corticosteroid sprays may be helpful.

### 5.2 Surgical options

Surgery can be performed on the septum, the inferior turbinates or the lateral nasal wall. Many patients with NVO and caudal septal deflection simply need a septoplasty and reduction of the inferior turbinates, particularly the anterior heads, to achieve a satisfactory outcome (13, 14). However, failure rates for septoplasty have been reported to be between 19% and 50% (15). One of the most common causes for patients to still report nasal obstruction after septoplasty is that NVO was not recognised pre-operatively (15, 16).

Operations on the lateral wall are more technically demanding, may have aesthetic implications and may not always be successful. Procedures may be directed specifically at either internal or external valve narrowing. Some aim to increase the cross-sectional area, while others attempt to strengthen or tension the lateral wall to reduce dynamic collapse.

Several techniques have been described to address lateral crus insufficiency at the level of the external nasal valve, and they aim to widen the dome angle and stabilise the lateral wall (17). Among these, grafts and suturing techniques are commonly employed. The techniques that address the internal nasal valve are usually done to widen the angle between the septum and the ULCs. This is done to either correct a static INV narrowing or to reduce the negative pressure at the level of the scroll area that determines the dynamic INV collapse. Spreader grafts are used to broaden the angle of the nasal valve up to the middle one-third of the nasal vault, and are the “gold standard” for INV reconstruction (12), particularly effective when placed under intact connections between the ULCs and the septum. Autospreader flaps are an alternative in which the patient's own ULCs are used as spreader grafts, avoiding the need to harvest grafts from other sites. Alar batten grafts, fashioned from septal or auricular cartilage, can be placed in a subcutaneous pocket caudally to the ULC or LLC, to stabilise the valve lateral wall. These grafts are mainly indicated for cases where excessive LLC resection has occurred, as they provide dynamic support to the lateral wall and correct cosmetic deformities in the supra-alar region. ULC splay graft, harvested from conchal cartilage, can be used to reconstruct the middle third of the nose, positioning the graft over the dorsum of the septum and beneath the ULCs. The butterfly graft (18, 19) represents an evolution of the splay graft, being placed superficially to the ULCs (after a dorsal hump reduction) and beneath the cephalic edge of the LLCs. Both techniques enlarge the angle of the INV angle and provide structural support to the ULCs. As a recent alternative, titanium butterfly graft implants show promising results for correcting INV insufficiency. Alar rim grafts are an option for external nasal valve collapse. The graft is placed within a pocket along the caudal border of the LLC, at the level of the lateral crus, extending to the alar-facial junction. It reinforces the alar rim, preventing collapse during breathing, and additionally providing subtle aesthetic improvement of the nostril symmetry and contour.

Besides grafts, simpler soft tissue procedures can reinforce the lateral wall of the INV. An example is the lateral crural J-flap, where a J-shaped chondrocutaneous incision is made over the anterior and inferior margins of the lateral crus of the lower lateral cartilage (14). A 2 mm sliver of mucosa and cartilage excised, and the wound edges approximated with sutures. It does not require harvesting cartilage grafts to support the valve, with the benefits of minimal cosmetic implications and is technically easy to perform. It can be used in cases of mild collapse of the lateral wall.

Multiple suturing techniques have been developed, including suspension sutures of LLC to the lower border of the orbit, and flaring sutures. However, long-term studies demonstrate a loss of efficacy over time (15, 20). One such study not only observed a low success rate in subjective improvement but also a high rate of complications, such as inflammation, pain under the eye, and the need for re-exploration of the area with resulting permanent scarring (14, 16). Suture-based techniques are therefore not recommended as first-line treatment.

More recent treatment options include bioabsorbable nasal valve implants (e.g., *Latera*) and radiofrequency to the nasal valve (e.g., *VivAer*). Implants mimic batten grafts to provide structural support to the upper and lower lateral cartilages, preventing their collapse. Via a minimally invasive procedure, both the implant and radiofrequency treatment stimulate a localized fibrotic response that will support the valve. The procedure has the great advantage of being performed in the office, but complications, including pain, abscess and implant protrusion, have been reported (21–26).

## 6 Proposed management algorithm

**Figure 3:**
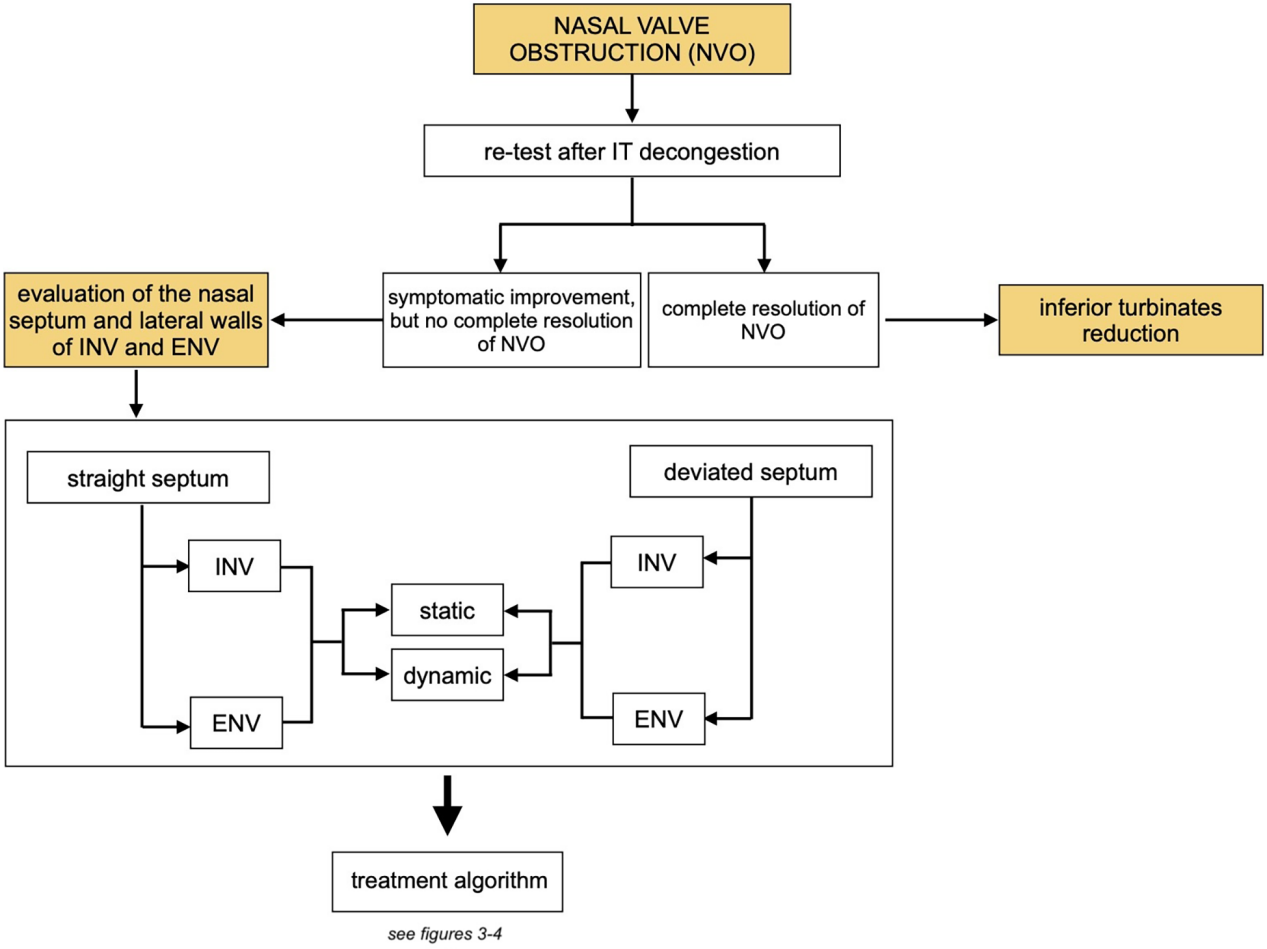
Overview of the treatment algorithm: assessment of the role of the inferior turbinates and subsequent management steps. (NVO, nasal valve obstruction; IT, inferior turbinates; INV, internal nasal valve; ENV, external nasal valve).

The difficulty in assessing NVO is that the valve is made of three different structures that can interchangeably or simultaneously be abnormal and need surgical correction (Figure 3). However, no current test specifically addresses each structure in isolation, eliminating the effect of the others.

**Figure 4:**
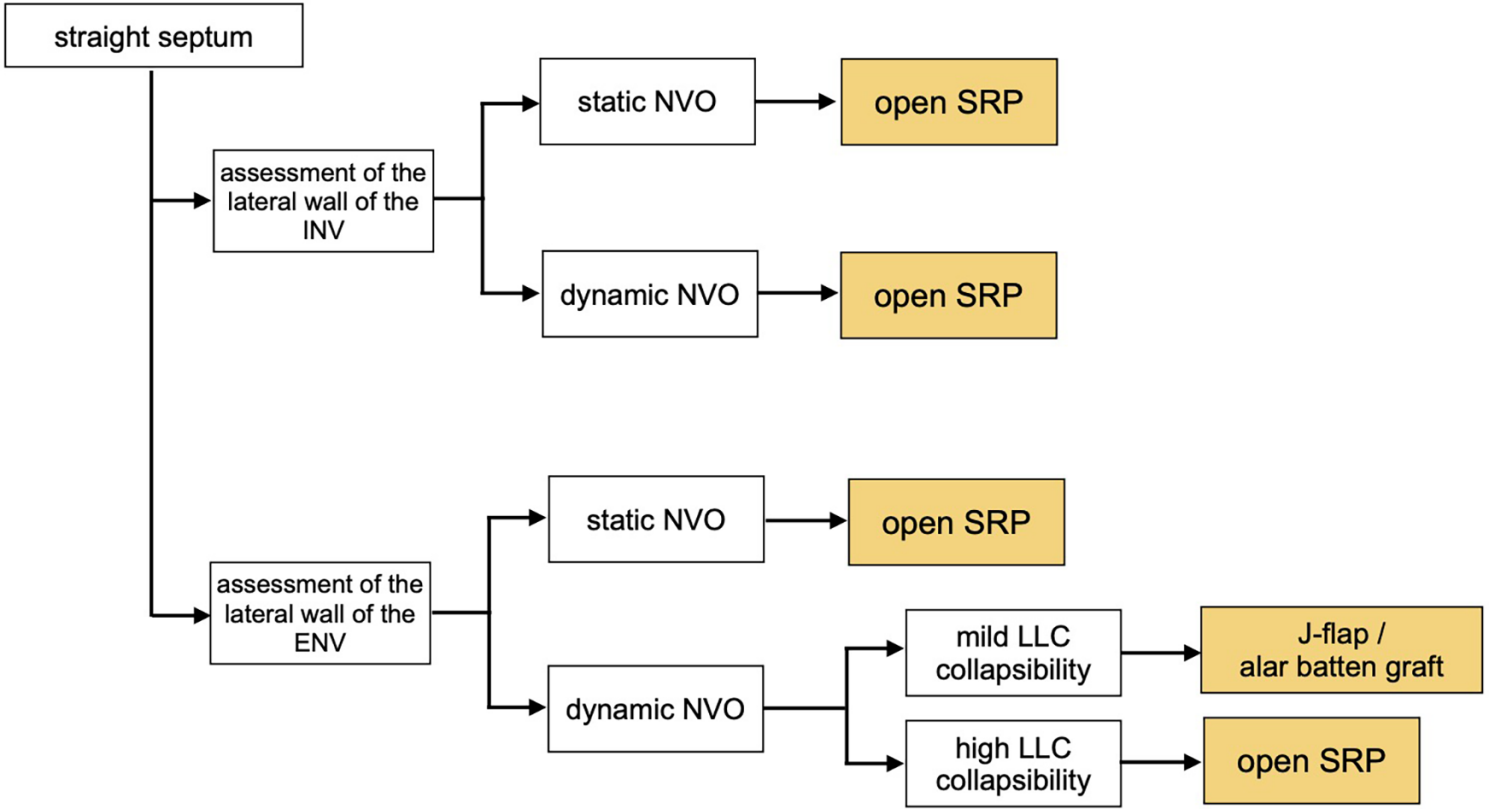
Treatment algorithm in cases with straight septum. The assessment of the lateral walls allows to determine if the obstruction is caused by a static narrowing of the upper/lower lateral cartilages, or by their dynamic collapse during inspiration. (INV, internal nasal valve; ENV, external nasal valve).

**Figure 5:**
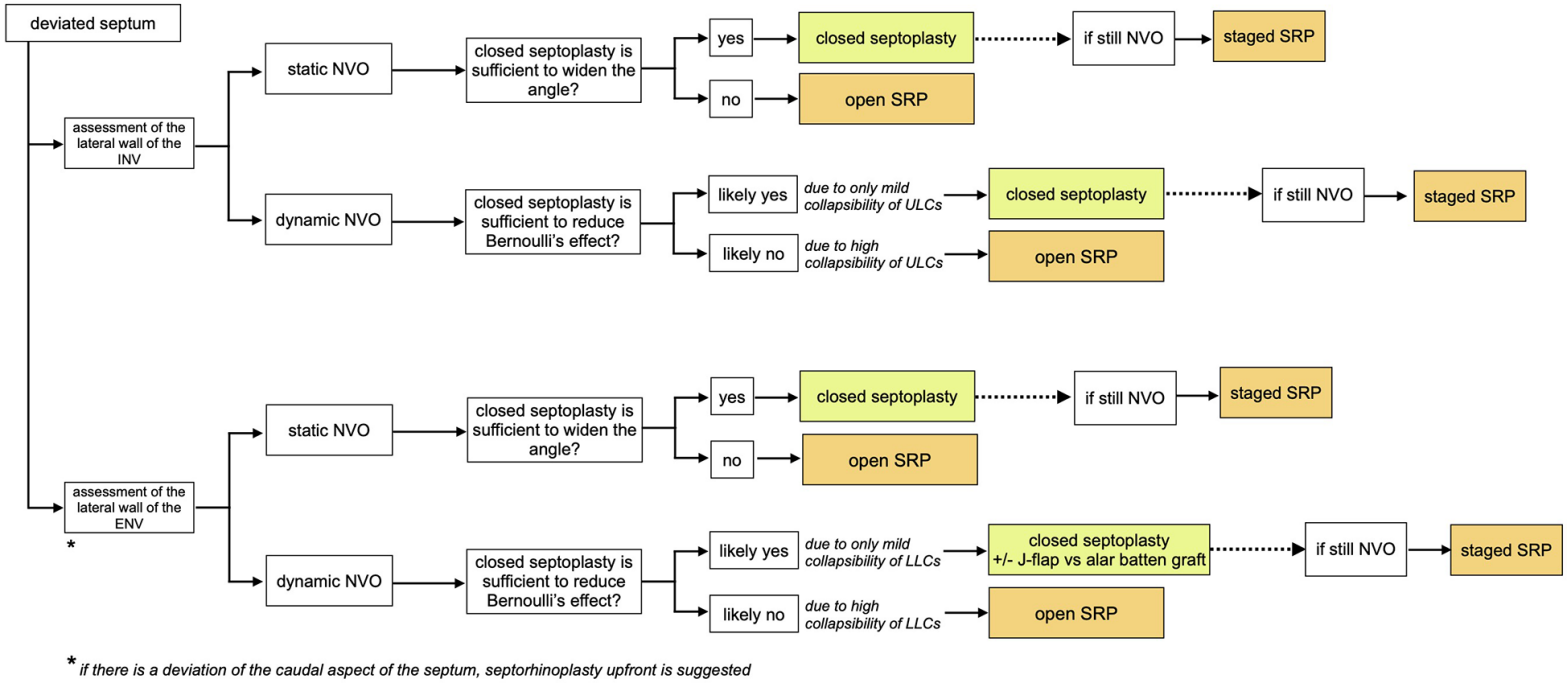
Treatment algorithm in cases with deviated septum. Once it is determined whether the valve narrowing is static or dynamic, surgical management is adjusted based on the effectiveness of closed septoplasty alone in resolving the obstruction. In dynamic NVO, this depends on the degree of collapsibility of the lateral cartilages; if the collapsibility is mild, a closed septoplasty may be sufficient to resolve NVO by reducing Bernoulli's effect.

The literature does not currently provide structured approaches to assessing and managing this condition. Many studies report retrospective case series, and treatment is often suggested based on expert opinions. We have created algorithms (Figures 3–5) to simplify the concepts that influence the treatment pathway of NVO.

The first key step is to determine the role of inferior turbinate in NVO. Even minor changes in the cross-sectional area of the nasal valve due to hypertrophy of the inferior turbinates can significantly increase resistance to airflow (17). Decongestion of the inferior turbinates can be used to test and diagnose if the reduction of the IT (especially at the level of the anterior head) lowers the resistance to airflow and is sufficient to improve a patient's symptoms, possibly making the surgical reduction of the inferior turbinates the only procedure needed in such cases.

The second key step is to determine the presence and role of septal deviation on NVO, as well as its relationship with the upper and lower lateral cartilages. Conservative management with nasal strips of nasal dilators could be a reasonable initial approach. If suboptimal symptom control, surgical repair of NVO is recommended.

In static NVO, the angle defined by the upper part of the nasal septum and the lateral walls of INV and ENV is narrow, yet no collapse of the lateral walls is present. In these cases, depending on the shape of the nasal septum and if it is the main contributing factor to the narrowing of the angle, a closed septoplasty may be sufficient. When the septal deviation is very high, especially at the level of the ENV, an open approach is suggested to reconstruct the septum. Likewise, an open approach is also recommended in cases where the cross-sectional narrowing of the valve is primarily due to the static narrowing of the lateral nasal cartilages, with or without septal deviation (27). Spreader or auto-spreader graft techniques are an example of surgical management for these types of cases.

If nasal obstruction is secondary to a dynamic collapse of the nasal valve, the focus should be on addressing the lateral wall. In the presence of a septal deviation and lateral cartilage collapse, patients may opt for a septorhinoplasty upfront or to undergo a septoplasty and determine the role of the lateral nasal wall in a staged manner. The latter is a valid option, especially when the valve lateral cartilages present with only mild collapsibility, and a concomitant septal deviation could be the main determinant of the lateral collapse due to Bernoulli's effect. Novel in-office minimally invasive procedures have limited evidence but may have a role, and crural J-flaps or alar batten grafts are also relatively simple methods of improving NVO (28). Otherwise, an open approach is required to reinforce the lateral nasal wall.

## 7 Conclusion

Nasal valve obstruction is a multifaceted condition arising from various combinations of anatomical factors. We elucidated the fundamental concepts underlying the pathophysiology of NVO, allowing patient stratification into a streamlined treatment algorithm. This study is limited by the significant variability within the existing literature, which primarily consists of retrospective case series, thereby limiting the level of evidence supporting specific interventions. Furthermore, the subjective nature of NVO and the reliance on patient-reported outcomes introduce variability in assessing treatment success. Efforts should be directed toward enhancing objective diagnostic methods, establishing standardized outcome measures, and conducting prospective studies to validate the effectiveness of diverse surgical interventions for NVO.
